# Association of FGFR3 and FGFR4 gene polymorphisms with breast cancer in Chinese women of Heilongjiang province

**DOI:** 10.18632/oncotarget.5850

**Published:** 2015-09-27

**Authors:** Yongdong Jiang, Shanshan Sun, Wei Wei, Yanlv Ren, Jing Liu, Da Pang

**Affiliations:** ^1^ Department of Breast Surgery, The Third Affiliated Hospital, Harbin Medical University, Harbin, China; ^2^ Department of Anesthesiology, The Second Affiliated Hospital, Harbin Medical University, Harbin, China

**Keywords:** breast cancer, FGFR, polymorphism, genetic susceptibility, Chinese

## Abstract

**Background:**

The fibroblast growth factor (*FGF*) receptor pathway is activated in many tumors. *FGFR2* has been identified as a breast cancer susceptibility gene. Common variation in other *FGF* receptors might also affect breast cancer risk. We carried out a case-control study to investigate associations of variants in *FGFR3* and *FGFR4* with breast cancer in women from Heilongjiang Province.

**Methods:**

SNP rs2234909 and rs3135848 in *FGFR3* and rs1966265 and rs351855 in *FGFR4* were successfully genotyped in 747 breast cancer patients and 716 healthy controls using the SNaPshot method. The associations between SNPs and breast cancer were examined by logistic regression. The associations between SNPs and disease characteristics were examined by chi-square tests or one-way ANOVA as needed.

**Results:**

The minor alleles of rs1966265 and rs351855 in *FGFR4* were strongly associated with breast cancer in the population, with odds ratios of 1.335 (95%CI = 1.154-1.545) and 1.364 (95%CI = 1.177-1.580), respectively. However, no significant associations were detected between other SNPs and breast cancer. Analyses of the disease characteristics showed that SNP rs351855 was associated with lymph-node-positive breast cancer with a dose-dependent effect of the minor allele (*P* = 0.008).

**Conclusions:**

SNPs rs1966265 and rs351855 in *FGFR4* were associated with breast cancer in a northern Chinese population.

## INTRODUCTION

Breast cancer is a complex disease and one of the most common malignancies in women worldwide. The incidence of female breast cancer in both developing and developed countries continues to rise [1]. Many studies have demonstrated that its etiology is associated with multiple genetic and environmental factors. Fibroblast growth factors (*FGFs*) and their signaling pathways appear to play significant roles, not only in normal development and wound healing but also in tumor development and progression [2]. Genome-wide association studies have identified an intronic variant in the *FGFR2* gene as a breast cancer susceptibility locus [3, 4]. Further, more studies have strongly suggested that *FGFR2* is a breast cancer susceptibility gene [5-8]. *FGFR2* belongs to the human fibroblast growth factor (*FGF*) and receptor family, which consists of genes that play critical roles in cancer development due to their angiogenic potential and direct enhancement of tumor growth [9]. The amino-acid sequence of *FGFR2* is highly conserved across all *FGF* receptors [10]. The other *FGF* receptor genes may also be implicated in the development of breast cancer. We hypothesized that common variants in other genes in the *FGF* pathway might also raise breast cancer risk, and we carried out this case-control study to identify associations between breast cancer risk and common variants in the *FGF* receptor genes *FGFR3* and *FGFR4* by genotyping selected tag-SNPs in women from Heilongjiang Province, northeast of China.

## RESULTS

### Subject characteristics

The 747 cases and 716 controls were similar with regard to age at interview, age at first birth and family history (Table 1). However, compared with controls, cases tended to have a higher BMI, an earlier age at menarche, and a longer period of breastfeeding. There was also a significant difference in the distribution of menopausal status between cases and controls.

**Table 1:** The demographic characteristics of 747 breast cancer cases and 716 healthy controls

Characteristics	Case (n=747)	Controls (n=716)	*P*
Age	49.91±10.28	49.55±10.61	0.508
Body mass index, BMI	24.03±3.44	23.32±3.13	<0.001
Age at menarche	15.37±1.80	15.61±1.94	0.015
Age at first birth	23.79±6.11	24.00±7.41	0.556
Breastfeeding duration	16.59±13.77	11.43±7.48	<0.001
Menopausal status			
Pre-menopausal	415 (55.6)	457 (63.8)	0.001
Post-menopausal	332 (44.4)	259 (36.2)	
Family history			
No	622 (83.3)	607 (84.8)	0.431
Yes	125 (16.7)	109 (15.2)	

Note: Data presented as the mean ± standard deviation or as number (% of total number)

### Association between SNPs and breast cancer risk

Four SNPs were selected in this case-control study: rs2234909 and rs3135848 in the *FGFR3* gene and rs1966265 and rs351855 in the *FGFR4* gene. The allele and genotype distributions for all SNPs in cases and controls were shown in Table 3. The genotype distributions of all SNPs in controls did not deviate from Hardy–Weinberg equilibrium (*P* > 0.05).

**Table 2:** Disease characteristics of the study population

Characteristics	Cases (%)
Clinic stage (UICC)	
0	29 (3.88)
1	300 (40.16)
2	203 (27.18)
3-4	143 (19.14)
Unknown	72 (9.64)
Tumor size (cm)	
TZ≤2 cm	583 (78.05)
TZ>2 cm	88 (11.78)
Unknown	76 (10.17)
Tumor type	
DCIS	29 (3.88)
IDC	641 (85.81)
Others	66 (8.84)
Unknown	11 (1.47)
Bloom-Richardson grade	
1	41 (5.49)
2	405 (54.22)
3	120 (16.06)
Unknown	181 (24.23)
LN involvement	
Positive	294 (39.36)
Negative	425 (56.89)
Unknown	28 (37.48)
ER status	
Positive	447 (59.84)
Negative	244 (32.66)
Unknown	56 (7.50)
PR status	
Positive	372 (49.80)
Negative	319 (42.70)
Unknown	56 (7.50)
HER2 status	
Positive	119 (15.93)
Negative	492 (65.86)
Unknown	136 (18.21)
P53 status	
Positive	185 (24.77)
Negative	499 (66.80)
Unknown	63 (8.43)
Ki67 status	
Positive	428 (57.30)
Negative	258 (34.54)
Unknown	61 (8.17)
Intrinsic subtypes	
Luminal A	149 (19.95)
Luminal B	273 (36.55)
HER2-positive	162 (21.69)
Triple-negative	27 (3.61)
Unknown	136 (18.20)

Note: DCIS: ductal carcinoma in situ, IDC: infiltrating duct carcinoma, LN: lymph node, TZ: tumor size, ER: estrogen receptor, PR: progesterone receptor, HER2: human epidermal growth factor receptor 2.

**Table 3:** Genotype distributions, odds ratios (OR) and 95% confidence intervals (CI) for the association between breast cancer susceptibility loci of FGFR3 and FGFR4 in 747 breast cancer cases and 716 controls

Genotype	Case (n=747)	Control (n=716)	OR (95%CI)	*P*	OR (95%CI)^a^	*P*^a^
rs1966265						
AA	168 (22.5)	226 (31.6)	1.000		1.000	
AG	408 (54.6)	364 (50.8)	1.508 (1.181-1.926)	0.001	1.581 (1.232-2.028)	3*10^−4^
GG	171 (22.9)	126 (17.6)	1.826 (1.346-2.476)	1*10-4	1.890 (1.387-2.576)	5*10^−5^
G allele	750 (50.2)	616 (43.0)	1.335 (1.154-1.545)	1*10-4	1.360 (1.173-1.577)	5*10^−5^
AG+GG	579 (77.5)	490 (68.4)	1.590 (1.259-2.007)	9*10-5	1.661 (1.310-2.106)	3*10^−5^
rs351855						
GG	205 (27.4)	270 (37.7)	1.000		1.000	
GA	404 (54.1)	348 (48.6)	1.529 (1.213-1.927)	3*10-4	1.555 (1.230-1.967)	2*10^−4^
AA	138 (18.5)	98 (13.7)	1.855 (1.352-2.544)	1*10-4	1.899 (1.377-2.617)	8*10^−5^
A allele	680 (45.5)	544 (38.0)	1.364 (1.177-1.580)	4*10-5	1.382 (1.190-1.605)	2*10^−5^
GA+AA	542 (72.6)	446 (62.3)	1.601 (1.284-1.996)	3*10-5	1.631 (1.303-2.040)	2*10^−5^
rs2234909						
TT	672 (90.0)	638 (89.1)	1.000		1.000	
TC	71 (9.5)	77 (10.8)	0.875 (0.623-1.230)	0.443	0.903 (0.639-1.276)	0.563
CC	4 (0.5)	1 (0.1)	3.798 (0.423-34.068)	0.233	3.884 (0.427-35.290)	0.228
C allele	79 (5.3)	79 (5.5)	0.956 (.694-1.318)	0.784	0.985 (.712-1.364)	0.929
TC+CC	75 (10.0)	78 (10.9)	0.913 (0.653-1.276)	0.594	0.942 (0.670-1.324)	0.731
rs3135848						
TT	576 (77.1)	553 (77.2)	1.000		1.000	
TC	157 (21.0)	155 (21.6)	0.972 (0.757-1.250)	0.827	0.971 (0.753-1.252)	0.821
CC	14 (1.9)	8 (1.1)	1.680 (0.699-4.036)	0.246	1.619 (0.658-3.981)	0.294
C allele	185 (12.4)	171 (11.9)	1.042 (.835-1.301)	0.715	1.035 (.826-1.296)	0.767
TC+CC	171 (22.9)	163 (22.8)	1.007 (0.789-1.286)	0.954	1.002 (0.782-1.284)	0.985

a Adjusted for age, BMI, age at menarche and menopausal status.

The results showed that the minor allele of rs1966265 and rs351855 in the *FGFR4* gene were strongly associated with breast cancer in Chinese women of Heilongjiang Province, with ORs of 1.335 (95%CI = 1.154-1.545) and 1.364 (95%CI = 1.177-1.580), respectively. We further analyzed the effect of the genotypes of these SNPs under three different genetic models (Table 3). After adjusting for age, BMI, age at menarche and menopausal status, for rs1966265, the AG and GG genotypes conferred a significantly increased risk for breast cancer compared to the AA genotype in the dominant model (OR = 1.661, 95%CI = 1.310-2.106, *P* = 3 × 10^−5^). For rs351855, the GA and AA genotype also significantly increased breast cancer risk compared to the GG genotype in the dominant model (OR =1.631, 95%CI = 1.303-2.040, *P* = 2 × 10^−5^). These results still showed statistical significance after Bonferroni correction.

Compared with the rs2234909 TT genotype, the TC and CC genotype showed a possible decreased risk for breast cancer in the dominant model (OR = 0.913, 95%CI = 0.653-1.276, *P* = 0.594). Compared with the rs3135848 TT genotype, the TC and CC genotype possibly conferred increased risk for breast cancer in the dominant model (OR = 1.007, 95%CI = 0.789-1286, *P* = 0.954).

### Stratified analysis by age, BMI, age at menarche and menopausal status

The results of stratified analyses are shown in Supplement Table 1. For the patients whose age was no more than 50, the minor allele of both rs1966265 and rs351855 significantly increased breast cancer risk under co-dominant and dominant models (adjusted *P* < 0.05). For the patients whose age was greater than 50, in the dominant model, combined genotypes (AG+GG) of rs1966265 had a 1.597-fold increase breast cancer risk compared with the genotype AA (adjusted OR = 1.597, 95% CI = 1.123-2.272, *P* = 0.009). For rs351855, the carriers with GA and AA genotypes had a similar breast cancer risk (adjusted OR = 1.577, 95% CI = 1.122-2.216, *P* = 0.009). Both rs1966265 and rs351855 were still associated with breast cancer risk under co-dominant and dominant models when stratified by BMI, age at menarche and menopausal status (corrected *P* < 0.05).

### Associations between SNPs and breast cancer characteristics

We then analyzed the effects of these SNPs on a series of disease characteristics in the patient cohort, including lymph node metastasis, tumor size, tumor grade, clinic stage, and the status of estrogen receptor (ER) or progesterone receptor (PR), HER2, P53, Ki67, and intrinsic subtypes (Luminal A, Luminal B, HER2-positive, and Triple-negative) (Supplement Table 2).

For SNP rs351855 in the *FGFR4* gene, it was found that the patients with genotypes GA and AA were more likely to have lymph-node-positive tumors compared to the patients with genotype GG (*P* = 0.008). Furthermore, we observed a dose-dependent effect of the A risk allele; each additional copy increased the probability of lymph node metastasis. For SNP rs1966265, the patients with AG and GG genotypes had a trend toward having lymph-node-positive tumors compared to the patients with genotype AA (*P* = 0.066). We did not find associations for the two SNPs with other disease characters, including tumor size, tumor grade, clinic stage, and the status of ER or PR, HER2, P53, Ki67, and intrinsic subtypes. Additionally, in this study, there were no significant associations between rs2234909 and rs3135848 in the *FGFR3* gene and all disease characters.

## DISCUSSION

In this study, we genotyped two polymorphisms in the *FGFR4* gene, rs1966265 and rs351855, and two polymorphisms in the *FGFR3* gene, rs2234909 and rs3135848, and evaluated their association with breast cancer risk in women from Heilongjiang Province, northeast of China. We found that SNPs rs1966265 and rs351855 in the *FGFR4* gene could increase breast cancer risk in northern Chinese women, especially for lymph-node-positive breast cancer.

The *FGFR4* gene is located at chromosome 5q35–qter. Several studies have shown that *FGFR4* polymorphisms are associated with the progression of various tumor types, such as breast, colon, prostate, and sarcoma tumors [11-16]. SNP rs1966265 in the *FGFR4* gene is a missense variant. A consistent result from the Breast Cancer Association Consortium (BCAC) showed that rs1966265 increased breast cancer risk for Europeans and Asians [10]. The estimated OR per risk (G) allele was 1.03 (95%CI= 1.01-1.05; *P* = 0.006) for European women and 1.08 (95%CI = 1.03-1.14; *P* = 0.004) for Asian women. The authors in BCAC thought that the power was much lower for Asian and African–American women, and certainly required independent replication. Our study showed that the G allele of rs1966265 increased a 1.360-fold risk for breast cancer in northern Chinese women, which was higher than the BCAC results. Our results were consistent with the previous study. We also found that the G allele of rs1966265 had a possible trend of a correlation with lymph node metastasis in the breast cancer patients.

The Arg388Gly polymorphism, in which glycine is substituted for arginine (G388R) at codon 388, is an important polymorphism of the *FGFR4* gene. It corresponds to the SNP rs351855 in the dbSNP database (www.ncbi.nlm.nih.gov/SNP) [17, 18]. We found that it increased breast cancer susceptibility in northern Chinese women. Additionally, patients carrying the minor allele were more likely to have lymph-node-positive breast cancer compared to carriers with the major allele. This result was consistent with previous studies in other ethnic populations. Bange et al. found that minor allele carriers were overrepresented in a subset of patients with lymph-node-positive breast cancer, and the presence of rs351855 was linked to early disease relapse [17]. Seitzer et al. showed that the oncogenic potential of SNP rs351855 was greater in mammary tumors compared with mice with the wild-type SNP, and the development of pulmonary metastases occurred at an earlier stage[19]. A relationship between the missense mutations in *FGFR4* and poorer prognosis in lymph-node-positive breast cancer was also demonstrated. Thussbaset et al. found that this mutation played a role in the resistance to adjuvant systemic therapy because knockdown of *FGFR4* increased sensitivity to chemotherapeutic agents and attenuated growth [12]. The homozygous carriers for the major allele of rs351855 have been proposed to have important tumor suppressive functions that are carried out via the regulation of genes controlling invasion and motility, e.g., *MMP1*, suggesting that loss of the wild-type receptor would adversely affect disease progression [20].

The *FGFR3* gene, which is located on chromosome 4p16.3, comprises 19 exons and 18 introns, spanning 16.5 kb [21, 22]. Previous studies found that *FGFR3* gene mutations were associated with many epithelial malignancies, including cervical carcinoma, nasopharyngeal carcinoma, colorectal cancer and bladder cancer [23-26]. They also found that *FGFR3* mutant tumors were associated with a good prognosis [25]. Multivariate analysis of all the superficial tumors did establish that *FGFR3* mutations were associated with an increased risk of recurrence [25]. In our study, we did not find that SNP rs2234909 and rs3135848 in the *FGFR3* gene were associated with breast cancer risk. We also did not find associations of these two SNPs with clinical pathological characteristics.

In conclusion, we evaluated the associations of four SNPs in the *FGFR3* and *FGFR4* genes with breast cancer in Chinese women from northeastern China and confirmed the associations of SNPs rs1966265 and rs351855 with breast cancer. The two SNPs were also associated with lymph-node-positive breast cancer. Although this study might provide new insights to understand the association of *FGFs* family with breast tumorigenesis and contribute to the early detection of breast cancer, these results await further confirmation by an ethnicity-matched larger study. Further studies are also needed to characterize the functional sequences that cause breast cancer.

## MATERIALS AND METHODS

### Subjects

A total of 1,463 individuals-747 breast cancer patients and 716 healthy controls-were included in this study. Patients with sporadic breast cancer were recruited from the Department of Breast Surgery at the Third Affiliated Hospital of Harbin Medical University. Breast cancer in these patients was diagnosed based on their surgical and pathological evaluation, and their disease information was obtained from their medical files (Tables 1 and 2). The control group consisted of women of Han origin living in Harbin, in northeastern China. The women in the control group, who had no history of cancer, were matched for age and ethnicity with the cancer patients. The participants were not genetically related within three generations. After providing informed consent, each participant was interviewed to collect detailed information on demographic characteristics (Table 1), and each provided 5 ml of venous blood. The study took place from September 2008 to December 2011 and was approved by the ethics committee of Harbin Medical University.

### SNP selection and genotyping

We performed a combined analysis of functional significance and Tag SNP strategies to select four potentially functional SNPs in the *FGFR3* and *FGFR4* gene from the dbSNP and HapMap databases. The minor allele frequencies (MAF) of these SNPs were greater than 5%, and the pair-wise r^2^ values were greater than 0.8. Genomic DNA was isolated from EDTA anti-coagulated whole blood using the AxyPrep Blood Genomic DNA Miniprep Kit (Axygen Biotechnology, Tewksbury, MA, USA). The SNaPshot SNP assay was carried out to detect the polymorphisms at the four SNP loci. The resulting data were analyzed with GeneMapper^TM^ 4.0 software (Applied Biosystems, Foster City, CA, USA). For quality control purposes, genotyping was performed without knowledge of the case/control status of the subjects, and a 5% random sample of cases and controls was genotyped twice by different persons; the reproducibility was 100%.

### Statistical analysis

The genotype frequencies were tested for Hardy–Weinberg equilibrium using the chi-square test among the controls. Differences between cases and controls in demographic characteristics and risk factors were evaluated by the chi-square test (for categorical variables) or Student's *t*-test (for continuous variables). Disease characteristics were compared with patient genotypes using the chi-square test (for categorical variables) or one-way ANOVA (for continuous variables). Associations between genotypes and breast cancer risk were estimated by computing odds ratios (ORs) and 95% confidence intervals (CIs) from logistic regression with adjustment for age, body mass index (BMI), age at menarche and menopausal status. Homozygotes for major allele were the reference group, and then heterozygotes and homozygotes for the minor allele were compared with the reference group, respectively. The dominant model was run with the homozygote for the minor allele and the heterozygote versus the reference group. All statistical tests were two-sided; a *P* value equal to or less than 0.05 was considered statistically significant, and a *P* value less than 0.1 was considered a possible trend that could be explored further in larger study groups. Statistical analyses were performed using SPSS for Windows (version 13.0; SPSS, Chicago, IL). The *P* value was adjusted for the four analyzed SNPs using Bonferroni correction, and a corrected *P* value < 0.013 (corrected α = 0.05/4) was considered statistically significant.

## SUPPLEMENTARY MATERIAL TABLES


