# Supplementary material for: Association of FGFR3 and FGFR4 gene polymorphisms with breast cancer in Chinese women of Heilongjiang province
Source: Oncotarget. 2015 Sep 27;6(32):34023–9. doi: 10.18632/oncotarget.5850 (PMC4741823; doi:10.18632/oncotarget.5850)
Supplement: Supplementary file 1 [file oncotarget-06-34023-s001.pdf]

Association of FGFR3 and FGFR4 gene polymorphisms with breast cancer in Chinese women of Heilongjiang province

Supplementary Material

Supplement Table1. Stratified analysis between *FGFR3* and *FGFR4* SNPs and breast cancer risk by age, BMI, age at menarche and menopausal status

	Age						BMI					
	≤50			>50			≤23			>23		
	Case/ controls	OR(95%CI)	P^a	Case/ controls	OR(95%CI)	P^a	Case/ controls	OR(95%CI)	P^a	Case/ controls	OR(95%CI)	P^a
rs2234909												
TT	372/363	1.000		300/275	1.000		265/292	1.000		407/346	1.000	
TC	39/42	0.933(0.588-1.480)	0.767	32/35	0.872(0.514-1.481)	0.613	23/44	0.577(0.338-0.986)	0.044	48/33	1.273(0.794-2.041)	0.317
CC	2/0	(-)	0.999	2/1	1.517(0.136-16.883)	0.735				4/1	4.192(0.460-38.218)	0.204
TC+CC	41/42	0.978(0.619-1.544)	0.924	34/36	0.894(0.533-1.501)	0.672	23/44	0.577(0.338-0.986)	0.044	52/34	1.352(0.852-2.146)	0.200
rs3135848												
TT	314/326	1.000		262/227	1.000		218/259	1.000		358/294	1.000	
TC	94/75	1.285(0.911-1.812)	0.153	63/80	0.668(0.453-0.985)	0.042	66/73	1.059(0.722-1.553)	0.769	91/82	0.921(0.656-1.294)	0.635
CC	5/4	1.305(0.342-4.982)	0.697	9/4	1.645(0.467-5.795)	0.439	4/4	1.211(0.295-4.978)	0.791	10/4	1.863(0.571-6.083)	0.303
TC+CC	99/79	1.286(0.918-1.801)	0.144	72/84	0.714(0.490-1.041)	0.080	70/77	1.067(0.734-1.551)	0.735	101/86	0.967(0.695-1.345)	0.841
rs1966265												
AA	82/119	1.000		86/107	1.000		60/102	1.000		108/124	1.000	
AG	226/214	1.588(1.129-2.235)	0.008	182/150	1.625(1.121-2.355)	0.010	169/173	1.746(1.183-2.576)	0.005	239/191	1.492(1.078-2.065)	0.016
GG	105/72	2.142(1.416-3.241)	<0.001	66/54	1.525(0.947-2.455)	0.083	59/61	1.771(1.089-2.882)	0.021	112/65	2.029(1.352-3.044)	0.001
AG+GG	331/286	1.731(1.249-2.399)	0.001	248/204	1.597(1.123-2.272)	0.009	228/234	1.752(1.206-2.546)	0.003	351/256	1.629(1.196-2.218)	0.002
rs351855												
GG	107/149	1.000		98/121	1.000		78/123	1.000		127/147	1.000	
GA	216/202	1.514(1.103-2.079)	0.010	188/146	1.651(1.156-2.357)	0.006	164/165	1.602(1.116-2.299)	0.011	240/183	1.547(1.135-2.109)	0.006

AA	90/54	2.312(1.517-3.524)	<0.001	48/44	1.335(0.803-2.218)	0.265	46/48	1.586(0.962-2.616)	0.070	92/50	2.187(1.430-3.344)	<0.001
GA+AA	306/256	1.686(1.248-2.279)	0.001	236/190	1.577(1.122-2.216)	0.009	210/213	1.599(1.130-2.260)	0.008	332/233	1.683(1.253-2.260)	0.001

Continued

	Age at menarche						Menopausal status					
	≤15			>15			Pre-menopausal			Post-menopausal		
	Case/ controls	OR(95%CI)	*P*[a]	Case/ controls	OR(95%CI)	*P*[a]	Case/ controls	OR(95%CI)	*P*[a]	Case/ controls	OR(95%CI)	*P*[a]
rs2234909												
TT	369/335	1.000		303/303	1.000		372/409	1.000		300/229	1.000	
TC	30/35	0.757(0.451-1.270)	0.291	41/42	1.045(0.654-1.670)	0.853	41/48	0.945(0.607-1.472)	0.804	30/29	0.833(0.477-1.453)	0.519
CC				4/1	3.911(0.431-35.480)	0.225	2/0			2/1	1.712(0.153-19.195)	0.663
TC+CC	30/35	0.757(0.451-1.270)	0.291	45/43	1.118(0.708-1.764)	0.633	43/48	0.991(0.640-1.535)	0.968	32/30	0.863(0.501-1.486)	0.594
rs3135848												
TT	310/285	1.000		266/268	1.000		319/363	1.000		257/190	1.000	
TC	79/78	0.911(0.638-1.301)	0.609	78/77	1.023(0.710-1.474)	0.903	91/88	1.198(0.860-1.669)	0.285	66/67	0.700(0.469-1.043)	0.080
CC	10/7	1.134(0.416-3.088)	0.806	4/1	6.199(0.619-62.111)	0.121	5/6	1.082(0.325-3.608)	0.898	9/2	2.543(0.511-12.667)	0.254
TC+CC	89/85	0.930(0.660-1.311)	0.678	82/78	1.072(0.747-1.538)	0.706	96/94	1.191(0.861-1.647)	0.290	75/69	0.756(0.512-1.115)	0.158
rs1966265												
AA	84/110	1.000		84/116	1.000		86/130	1.000		82/96	1.000	
AG	221/190	1.638(1.153-2.327)	0.006	187/174	1.596(1.116-2.284)	0.010	231/241	1.485(1.068-2.065)	0.019	177/123	1.828(1.242-2.693)	0.002
GG	94/70	1.865(1.216-2.861)	0.004	77/56	1.947(1.235-3.069)	0.004	98/86	1.749(1.172-2.608)	0.006	73/40	2.136(1.296-3.519)	0.003
AG+GG	315/260	1.700(1.216-2.377)	0.002	264/230	1.684(1.197-2.368)	0.003	329/327	1.555(1.135-2.131)	0.006	250/163	1.907(1.321-2.752)	0.001
rs351855												
GG	101/137	1.000		104/133	1.000		110/163	1.000		95/107	1.000	
GA	221/180	1.742(1.253-2.422)	0.001	183/168	1.451(1.034-2.036)	0.031	221/229	1.456(1.071-1.980)	0.016	183/119	1.814(1.250-2.631)	0.002
AA	77/53	2.075(1.335-3.227)	0.001	61/45	1.759(1.095-2.825)	0.020	84/65	1.935(1.290-2.905)	0.001	54/33	1.798(1.060-3.050)	0.030
GA+AA	298/233	1.817(1.326-2.490)	<0.001	244/213	1.516(1.097-2.094)	0.012	305/294	1.564(1.168-2.095)	0.003	237/152	1.810(1.270-2.580)	0.001

[a]Adjusted for age, BMI, age at menarche, and menopausal status (excluded the stratified factor in each stratum).

SNP: rs2234909 in *FGFR3*

Patient disease characteristics		n	Phenotype per genotype			P, three groups†	P, two groups‡
			AA	AB	BB		
Genotype(number of patients)		747	672	71	4		
Clinic stage(UICC)	0	675	4.8%	.0%	.0%	0.323	0.194
	1		43.5%	54.0%	33.3%		
	2		30.2%	27.0%	66.7%		
	3-4		21.5%	19.0%	.0%		
Tumor size(cm)	≤2	671	86.8%	87.3%	100.0%	0.791	0.801
	＞2		13.2%	12.7%	.0%		
Bloom-Richardson grade	1	566	7.5%	5.7%	.0%	0.761	0.825
	2		71.3%	71.7%	100.0%		
	3		21.2%	22.6%	.0%		
LN involvement (%)		719	41.5%	34.8%	50.0%	0.523	0.334
ER positive (%)		691	65.3%	56.9%	100.0%	0.136	0.334
PR positive (%)		691	54.0%	52.3%	50.0%	0.954	0.770
HER2 positive (%)		611	19.1%	22.4%	25.0%	0.803	0.515
Ki67 positive (%)		686	61.4%	72.3%	50.0%	0.199	0.119
P53 positive (%)		684	26.5%	32.3%	25.0%	0.603	0.340
Intrinsic subtypes	Luminal A	611	24.6%	22.4%	25.0%	0.828	0.959
	Luminal B		44.8%	41.4%	75.0%		
	HER2 positive		26.2%	31.0%	.0%		
	Triple negative		4.4%	5.2%	.0%		

SNP: rs3135848 in *FGFR3*

Patient disease characteristics		n	Phenotype per genotype			*P*, three groups†	*P*, two groups‡
			AA	AB	BB		
Genotype(number of patients)		747	576	157	14		
Clinic stage(UICC)	0	675	4.2%	5.2%	.0%	0.285	0.903
	1		44.1%	48.1%	16.7%		
	2		29.9%	29.6%	41.7%		
	3-4		21.8%	17.0%	41.7%		
Tumor size(cm)	≤2	671	86.9%	88.1%	75.0%	0.438	0.967
	＞2		13.1%	11.9%	25.0%		
Bloom-Richardson grade	1	566	6.8%	9.2%	7.7%	0.203	0.456
	2		72.7%	69.7%	46.2%		
	3		20.5%	21.1%	46.2%		
LN involvement (%)		719	41.7%	35.1%	71.4%	0.023	0.441
ER positive (%)		691	64.3%	64.8%	78.6%	0.544	0.691
PR positive (%)		691	53.5%	54.9%	57.1%	0.923	0.713
HER2 positive (%)		611	19.4%	19.7%	21.4%	0.980	0.900
Ki67 positive (%)		686	63.9%	55.7%	71.4%	0.160	0.127
P53 positive (%)		684	26.4%	28.6%	35.7%	0.669	0.490
Intrinsic subtypes	Luminal A	611	23.4%	29.5%	14.3%	0.503	0.667
	Luminal B		45.5%	39.3%	64.3%		
	HER2 positive		26.9%	26.2%	14.3%		
	Triple negative		4.2%	4.9%	7.1%		

SNP: rs1966265 in *FGFR4*

Patient disease characteristics		n	Phenotype per genotype			*P*, three groups†	*P*, two groups‡
			AA	AB	BB		
Genotype(number of patients)		747	168	408	171		
Clinic stage(UICC)	0	675	3.2%	5.2%	3.3%	0.212	0.763
	1		47.4%	44.8%	40.5%		
	2		29.5%	31.4%	27.5%		
	3-4		19.9%	18.6%	28.8%		
Tumor size(cm)	≤2	671	87.8%	86.8%	86.2%	0.910	0.693
	＞2		12.2%	13.2%	13.8%		
Bloom-Richardson grade	1	566	7.5%	7.6%	6.2%	0.801	0.981
	2		70.9%	70.0%	76.0%		
	3		21.6%	22.4%	17.8%		
LN involvement (%)		719	35.4%	39.9%	48.8%	0.041	0.101
ER positive (%)		691	63.1%	64.6%	66.7%	0.800	0.627
PR positive (%)		691	52.2%	52.5%	58.8%	0.373	0.646
HER2 positive (%)		611	20.4%	17.6%	23.1%	0.374	0.745
Ki67 positive (%)		686	58.7%	62.8%	65.1%	0.495	0.282
P53 positive (%)		684	24.5%	27.5%	28.5%	0.704	0.420
Intrinsic subtypes	Luminal A	611	21.8%	24.2%	27.6%	0.913	0.831
	Luminal B		47.2%	44.2%	43.3%		
	HER2 positive		26.1%	26.9%	26.1%		
	Triple negative		4.9%	4.8%	3.0%		

SNP: rs351855 in *FGFR4*

Patient disease characteristics		n	Phenotype per genotype			*P*, three groups†	*P*, two groups‡
			AA	AB	BB		
Genotype(number of patients)		747	205	404	138		
Clinic stage(UICC)	0	675	4.3%	4.7%	3.2%	0.702	0.669
	1		47.9%	43.8%	41.1%		
	2		29.3%	30.9%	29.0%		
	3-4		18.6%	20.7%	26.6%		
Tumor size(cm)	⩽2	671	87.7%	86.9%	85.5%	0.850	0.697
	＞2		12.3%	13.1%	14.5%		
Bloom-Richardson grade	1	566	8.3%	7.6%	4.8%	0.510	0.536
	2		68.2%	71.1%	78.1%		
	3		23.6%	21.4%	17.1%		
LN involvement (%)		719	33.3%	41.9%	49.6%	0.011	0.010
ER positive (%)		691	63.0%	65.7%	64.3%	0.817	0.569
PR positive (%)		691	52.1%	53.4%	57.9%	0.570	0.567
HER2 positive (%)		611	19.7%	17.9%	24.1%	0.369	0.945
Ki67 positive (%)		686	57.4%	63.6%	66.4%	0.209	0.093
P53 positive (%)		684	26.8%	27.0%	27.4%	0.994	0.940
Intrinsic subtypes	Luminal A	611	21.4%	25.5%	25.9%	0.694	0.309
	Luminal B		47.4%	43.6%	43.5%		
	HER2 positive		24.9%	27.0%	27.8%		
	Triple negative		6.4%	3.9%	2.8%		

Note: LN: lymph node, ER: estrogen receptor, PR: progesterone receptor, HER2: human epidermal growth factor receptor2.
A: major allele; B: minor allele.

†Comparing the three genotype groups separately.

‡A codominant model, comparing the group of homozygous patients for the major allele with the combined group of heterozygous patients and patients homozygous for the minor allele.
